# Prevalence of respiratory symptoms and associated factors among sanitation workers in Sub Saharan Africa: a systematic review and meta-analysis

**DOI:** 10.3389/fpubh.2025.1519209

**Published:** 2025-06-04

**Authors:** Gete Berihun, Belay Desye, Leykun Berhanu, Chala Daba, Zebader Walle, Abebe Kassa Geto

**Affiliations:** ^1^Department of Environmental Health, College of Medicine and Health Sciences, Debre Markos University, Debre Markos, Ethiopia; ^2^Department of Environmental Health, College of Medicine and Health Sciences, Wollo University, Dessie, Ethiopia; ^3^Department of Social and Public Health, College of Health Sciences, Debre Tabor University, Debre Tabor, Ethiopia; ^4^Department of Public Health, College of Health Sciences, Woldia University, Woldia, Ethiopia

**Keywords:** respiratory–epidemiology, systematic review and meta-analysis, risk factors, Sub Saharan Africa, prevalence, sanitation workers

## Abstract

**Introduction:**

Occupational health problems are the major issues of the world, particularly in developing countries. Sanitation workers are facing various health problems with little attention while conducting their day today activities. The review emphasizes the critical need for policies that ensure safety standards, provide proper protective gear, and establish regular health monitoring to protect workers’ health and wellbeing.

**Objective:**

The aim of the study was to assess the prevalence of respiratory symptoms and associated factors among sanitation workers in Sub-Saharan Africa.

**Methods and materials:**

This systematic review and meta-analysis was done based on the Preferred Reporting Items for Systematic Reviews and Meta-Analysis (PRISMA 2020). Literatures were searched using various database like Google scholar, Science-Direct, Pub-Med (Medline), Hinari, and Google. From eligible studies, data was extracted using Microsoft excel and exported to STATA version 14 statistical software for analysis. The prevalence of respiratory symptom was estimated using a random effect model. Publication bias was determined using Egger test and funnel plot whereas heterogeneity was evaluated using I^2^. Fortunately, 13 studies were included with a total participants of 4,401.

**Results:**

The pooled prevalence of respiratory symptoms among sanitation workers was 43.79% (95% CI: 35.26, 52.33; *I*^2^ = 97.3%, *p* < 0.000). Sanitation workers with a history of respiratory illness were 4.16 times more likely to have had respiratory symptoms compared to those without a history (OR: 4.16, 95% CI: 2.67, 5.66). Additionally, sanitation workers who did not wear nose/mouse masks were 2.36 times more likely to experience respiratory symptoms compared to their counterparts (OR: 2.36, 95% CI: 1.40, 3.32). Moreover, Sanitation workers with working experiences of greater than five were 1.81 times more likely to experience respiratory symptoms than those with less than 5 years working experiences (OR: 1.81, 95% CI: 1.26, 2.39).

**Conclusion:**

Generally, half of the sanitation workers experienced respiratory symptoms. The symptoms were associated with history of respiratory illness, utilizing of nose/ mouth face mask and working experience. Hence, awareness creation session should conducted mainly for those with history of respiratory illness and poor personal protective users.

## Introduction

Sanitation is the measures taken to maintain health and prevent disease by ensuring clean and hygiene facilities and services which includes cleaning dirty toilets, septic tanks, streets, roads, and beaches (1). Currently, sanitation is highly linked with the attractiveness of a city (2, 3). Sanitation workers are at a considerable high risk for respiratory symptoms because of the nature of there working environment. The risk factors for the increment of the problem includes exposure to harmful biological and chemical agents, poor working conditions like insufficient ventilation, inadequate use of personal protective equipment resulting from lack of training, and low levels of health literacy (4–6).

Sanitation workers are exposed to a higher concentration of contaminants like bioaerosols, volatile organic compounds (VOCs), polycyclic aromatic hydrocarbons (PAHs), heavy metals, dioxins, and furans which are known toxicants for human health and environmental pollution. These toxicants may cause injuries, infections, musculoskeletal disorders, gastrointestinal issues, respiratory ailments, skin conditions, irritation of the nose and eyes, fatigue, headaches, allergies, and psychological impairment (1–3, 7, 8). The increased vulnerability of sanitation workers are highly exacerbated by poverty, illness, inadequate nutrition, substandard housing, child labor, migration, substance abuse, discrimination, social stigma, and, inadequate occupational health and safety measures societal neglect (6, 9).

The exact figure of sanitation workforce is unclear, however they are often the most overlooked and undervalued members of society (10). Globally, an estimated 56 million people, including 15 million in developing countries, works in unsanitary conditions while performing their routine activities (11). Sanitation workers faced various issues like unstable jobs with low wages, lack of robust legal protections, standard operating procedures, and policies to safeguard their rights (1). The International Labor Organization (ILO) estimates that over 125 million workers experience occupational accidents and 2.2 million deaths recorded each year (12). The European Union revealed that the economic cost of all work-related health issues amounts to between 2.6 and 3.8% of GDP (13).

Globally, 3.3 billion people rely on on-site sanitation systems which collects fecal sludge that need regular maintenance in its journey. Despite sanitation workers have a remarkable role in achieving Sustainable Development Goal (SDG) 6.2, they remain one of the most vulnerable groups, often lacking protective equipment, job security, and adequate access to preventive and remedial healthcare or social protections (9, 14).

Globally, the burden of respiratory problem accounts one-tenth of the non-communicable diseases with three-quarters of related deaths occurring in developing countries, mainly in Africa (15). The vulnerability of sanitation workers has been a longstanding global issue, particularly affecting those in low- and middle-income countries. These workers, who frequently operate within the informal economy, encounter not only occupational and environmental health risks but also difficulties in accessing healthcare, legal protections, and financial stability (4, 9, 16). Globally, respiratory diseases lead to approximately 4 million deaths and around 13,000 fatalities are attributed to work-related lung diseases and cancers, primarily resulting from exposure to chemicals and dust in the workplace each year (17). Generally, occupational respiratory diseases represent a major public health issue, constituting one-third of all reported work-related diseases and 10 to 20% of global deaths with sever problem is reported in low- and middle-income countries (LMIC) (7). The burden of respiratory symptom affected by various factors including age (15), BMI, sleeping condition (18), educational status of workers, interaction with pets, ventilation system of the house (19), energy utilization status (20), smoking habit (21), utilization of PPEs (20), work experience, working hours per day (15), and history of respiratory illness (19). Sanitation workers have a low economic and education status which may cause them to give little attention to pay for their health (2). A significant obstacle in providing support to sanitation workers is the lack of understanding regarding their profiles, including their specific needs, the challenges they encounter, and the conditions of their work environments (22). They have little influence over policy formulation and resource allocation at the national and local levels, which makes it difficult for them to improve their access to occupational safety and health (23). Despite there are some primary studies on the prevalence’s and associated factors among sanitation workers, there is no review done in SSA. Therefore, this review aimed to assess the prevalence of respiratory symptoms and associated factors among sanitation workers in SSA.

## Methods and materials

### Study setting

The study was conducted in SSA.

### Protocol and registration

The protocol for this systematic review was registered with the International Prospective Register of Systematic Reviews (PROSPERO), managed by the University of York Centre for Reviews and Dissemination. The protocol of the review was registered on December 27, 2023, under the protocol number CRD42023494212.

### Information search and search strategies

This review was done following the Preferred Reporting Items for Systematic Reviews and Meta-Analyses (PRISMA-2020) guideline. (S1) A comprehensive search was performed using various databases, including Science-direct, Hinari, Google scholar, Pub-Med (Medline) to identify relevant literatures. Additionally, a Google search was carried out to identify articles which were not indexed in the above mentioned databases. Furthermore, we also reviewed the reference lists of relevant studies and consulted with content experts to identify additional gray literature pertinent to this review. For the PubMed/MEDLINE database search, we used a combination of key terms and Boolean operators (AND, OR) to construct a detailed and comprehensive search strategy (“respiratory symptoms” OR (“acute respiratory symptoms” OR (“chronic respiratory symptoms” OR (“pulmonary symptoms” OR (“respiratory distress” OR (“lung-related symptoms” OR (“respiratory problem”) AND (“sanitation workers” OR “waste management workers” OR “garbage collectors” OR “Refuse collectors” OR “street sweepers” OR “land fill operators” OR “sewage treatment operators” OR cleaners OR janitors AND (“associated factors” OR “contributing factors” OR “determining factors” OR “risk factors” AND (“Angola” OR “Benin” OR “Botswana” OR “Burkina Faso” OR “Burundi” OR “Cabo-Verde” OR “Cameroon” OR “Central African Republic” OR “Chad” OR “Comoros” OR “Democratic Republic of the Congo” OR “Djibouti” OR “Equatorial Guinea” OR “Eritrea” OR “Eswatini” OR “Ethiopia” OR “Gabon” OR “Gambia” OR “Ghana” OR “Guinea” OR“Guinea-Bissau” OR “Ivory Coast” OR “Kenya” OR “Lesotho” OR “Liberia” OR “Madagascar” OR “Malawi” OR “Mali” OR “Mauritania” OR “Mauritius” OR “Mozambique” OR “Namibia” OR “Niger” OR “Nigeria” OR “Republic of the Congo” OR “Rwanda” OR “São Tomé and Príncipe” OR “Senegal” OR “Seychelles” OR “Sierra Leone” OR “Somalia” OR “South Africa” OR “South Sudan” OR “Sudan” OR “Tanzania” OR “Togo” OR “Uganda” OR “Zimbabwe.” The search was conducted from March to July 5, 2024.

### Eligibility criteria

**I. Inclusion Criteria:** Articles that met the following criteria were included in the review.

**II. Population:** The study participants consisted of workers employed as waste management workers, garbage collectors, refuse collectors, street sweepers, landfill operators, sewage treatment plant operators, cleaners, and janitors.

**III. Outcome Variables:** The outcome variables of this study was the prevalence of respiratory symptoms with the option of (present/absent) and/or associated factors.

**IV. Study Design:** This review encompasses cross-sectional study designs.

**V. Study Settings:** The research was conducted in Sub-Saharan Africa (SSA).

**VI. Language:** this review includes full text articles published only in English language.

**VII. Publication Period:** Articles published up to July 5, 2024, were included.

### Exclusion criteria

Qualitative studies, systematic reviews, letters to the editor, short communications, commentaries, and articles that could not be fully accessed after three attempts to reach the corresponding author were excluded from the review. Additionally, office cleaners, along with hotel and restaurant cleaners, were not part of the study.

### Study selection

GB and LB, the two independent reviewers, evaluated the titles, abstracts, and full texts of the articles to determine their eligibility based on the predetermined set of criteria. Articles considered eligible by both GB and LB were grouped together. If there was a disagreement between the two reviewers, a third independent reviewer, BD, was consulted to assist in making the final decision on whether to include or exclude the article.

### Data extraction and management

A standardized data extraction format was used to collect pertinent data from eligible studies. The data includes the author’s name, publication year, country, data collection method, sampling technique, sample size, prevalence of respiratory symptoms, and assessment of bias risk. EndNote reference management software was utilized to organize the search results and remove duplicate articles.

### Quality assessment of studies

The Joanna Briggs Institute (JBI) quality assessment tools for analytical cross-sectional studies were utilized to evaluate the quality of the articles included in the review (24). The assessment was based on several indicators, with response options of yes, no, unclear, and not applicable: (1) inclusion and exclusion criteria; (2) description of the study subjects and settings; (3) use of a valid and reliable method to measure exposure; (4) standard criteria for measuring the condition; (5) identification of confounding factors; (6) development of strategies to address confounding factors; (7) use of a valid and reliable method to measure outcomes; and (8) application of appropriate statistical analysis. The risk of bias was classified as low (scores of 6–8), moderate (3–5), and high (0–2), with articles demonstrating moderate and low risks of bias included in the final review (Supplementary information 2).

### Outcome of interest

This review has two outcome variables. The first one is the pooled prevalence of respiratory symptoms while the second outcome variable of this review was determination of risk factors of respiratory symptoms among sanitation workers in SSA using OR with 95% confidence interval (CI).

### Operational definitions

Sanitation workers: These are workers engaged in activities like sweeping streets, collecting waste from residences and public spaces, cleaning latrines and pits, maintaining toilets in schools and public areas, servicing restrooms in municipal, government, and private establishments, operating waste collection vehicles, managing fecal sludge, emptying septic tanks, cleaning sewers and manholes, overseeing sewage treatment plants, and handling wastewater and sludge at these sites (1).

Respiratory symptoms: a respiratory symptom is defined as the presence of 1 or more of the following symptoms such as chronic cough, chronic phlegm, chronic wheezing, and chronic chest tightness that lasted at least 3 months in a year (25, 26).

### Statistical method and analysis

Data were extracted using a Microsoft Excel spreadsheet and then imported into STATA version 14 for further analysis. The heterogeneity of the eligible studies was assessed using the I^2^ statistic, with cutoff points of 25–50%, 50–75, and >75% indicating low, moderate, and high heterogeneity, respectively (27). The pooled prevalence of respiratory symptoms among sanitation workers was estimated using the meta-prop command in STATA version 14. Subgroup analyses was done using variables on publication year, country, sample size, geographic location, and Types of sanitation workers (municipal waste collector vs. E-waste collectors). Sensitivity analyses was performed to evaluate the effect of each study on the overall pooled prevalence of respiratory symptoms. Publication bias was assessed using the funnel plot test and Egger’s regression test, with a *p*-value <0.05 at a 95% CI considered statistically significant (28). The results of the finding are presented using graphs, tables, text, and a forest plot based on the nature of the data.

## Results

### Searching process

**Figure 1:**
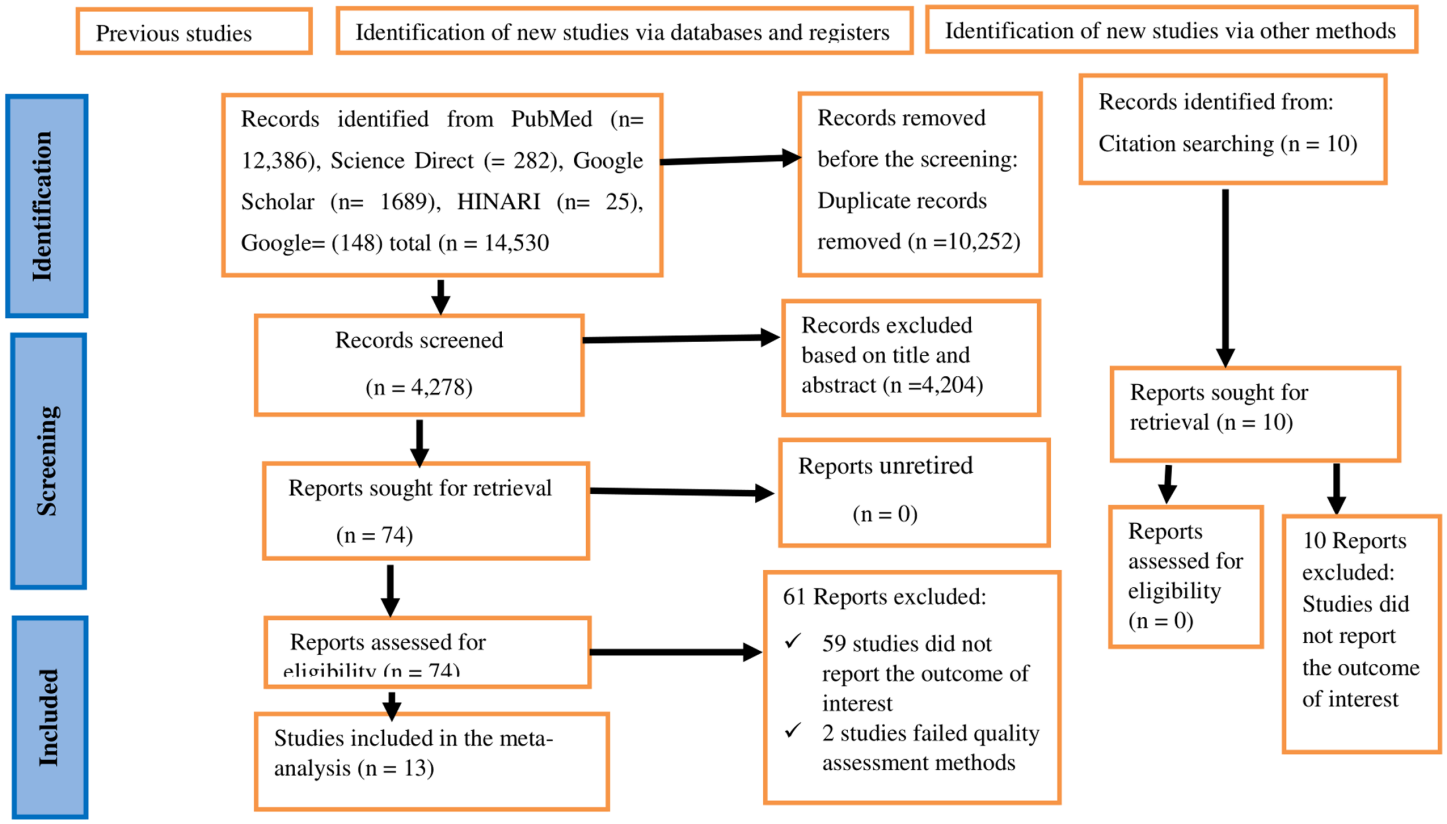
A PRISMA flow chart showing study selection for systematic review and meta-analysis on the prevalence of respiratory symptoms and associated factors among sanitation workers in SSA.

Extensive literature search was carried out across various databases and resulted 14,530 studies. After eliminating 10,252 duplicate articles, 4,278 studies were screened based on their titles and abstracts. Out of these, 4,204 were excluded for not meeting the established inclusion criteria. The remaining 74 articles were then evaluated for full-text eligibility. Following this review, an additional 61 studies were excluded—59 articles did not report the desired outcome, and 2 were deemed low quality. Ultimately, 13 studies were included in this review (29) (Figure 1).

### Characteristics of studies included in the review

In this review, a total of 13 studies were included in the final model and all of them were done by a cross-sectional study. The total sample size across the studies was 4,401, with sample size ranging from 137 to 718. These studies were published between 2012 and 2022 almost all of these studies utilized interviewer-based questionnaires and observational checklist as primary method of data collection. Based on study setting, 7 studies were from Ethiopia (7, 20, 25, 26, 30–32), 2 each from Nigeria (16, 33), two studies from Benin and Ivory Coast (34, 35), Sudan (36) and South Africa (37). The overall characteristics of studies included in this review are presented in the table below (Table 1).

**Table 1:** Descriptive summary of studies included determination of the prevalence of respiratory symptoms among sanitation workers in SSA in 2024.

Author, publication year	Study country	Publication year	Sampling technique	Method of data collection	Sample size	Prevalence	Risk of bias
Worede (2021) (7)	Ethiopia	2021	Simple random	Interviewer based questionnaire	391	35.3	Low
Eneyew (2021) (20)	Ethiopia	2021	Simple random	Pretested structure questionnaire &on the spot direct observational checklist	168	42.85	Low
Emiru (2017) (25)	Ethiopia	2017	Consecutive	Observational checklist and structured questionnaire	518	40.7	Low
Johnson (2020) (16)	Nigeria	2020	Simple random	Interviewer administered semi-structured questionnaire	150	47.3	Low
Muhammad (2020) (33)	Nigeria	2020	Convenience sampling	Self-administered questionnaire	129	60	Low
Tlotleng (2019) (37)	South Africa	2019	Convenience sampling	Interviewer-based questionnaire	361	58.5	Low
Manaye (2022) (30)	Ethiopia	2022	Convenience sampling	Interviewer-based questionnaire	392	45.4	Low
Tamene (2017) (26)	Ethiopia	2017	Simple random	Structured questionnaire	405	68.9	Low
Houngbégnon (2022) (34)	Benin & Ivoricost	2022	Simple random	Interviewer-based questionnaire	308	20.1	Low
Wachinou (2022) (35)	Benin	2022	Simple random	Interviewer-based questionnaire	148	33.1	Moderate
Melaku (2020) (31)	Ethiopia	2020	Multi-stage sampling	Structured interviewer administered questionnaire	576	22.6	Low
Makki (2022) (36)	Sudan	2022	Stratified random sampling	Standardized pre-tested questionnaire	718	38	Low
Gebremedhn (2019) (32)	Ethiopia	2019	Simple random	Structured interviewer administered questionnaire	137	58.39	Moderate

### Pooled prevalence of respiratory symptoms among sanitation workers in SSA

**Figure 2:**
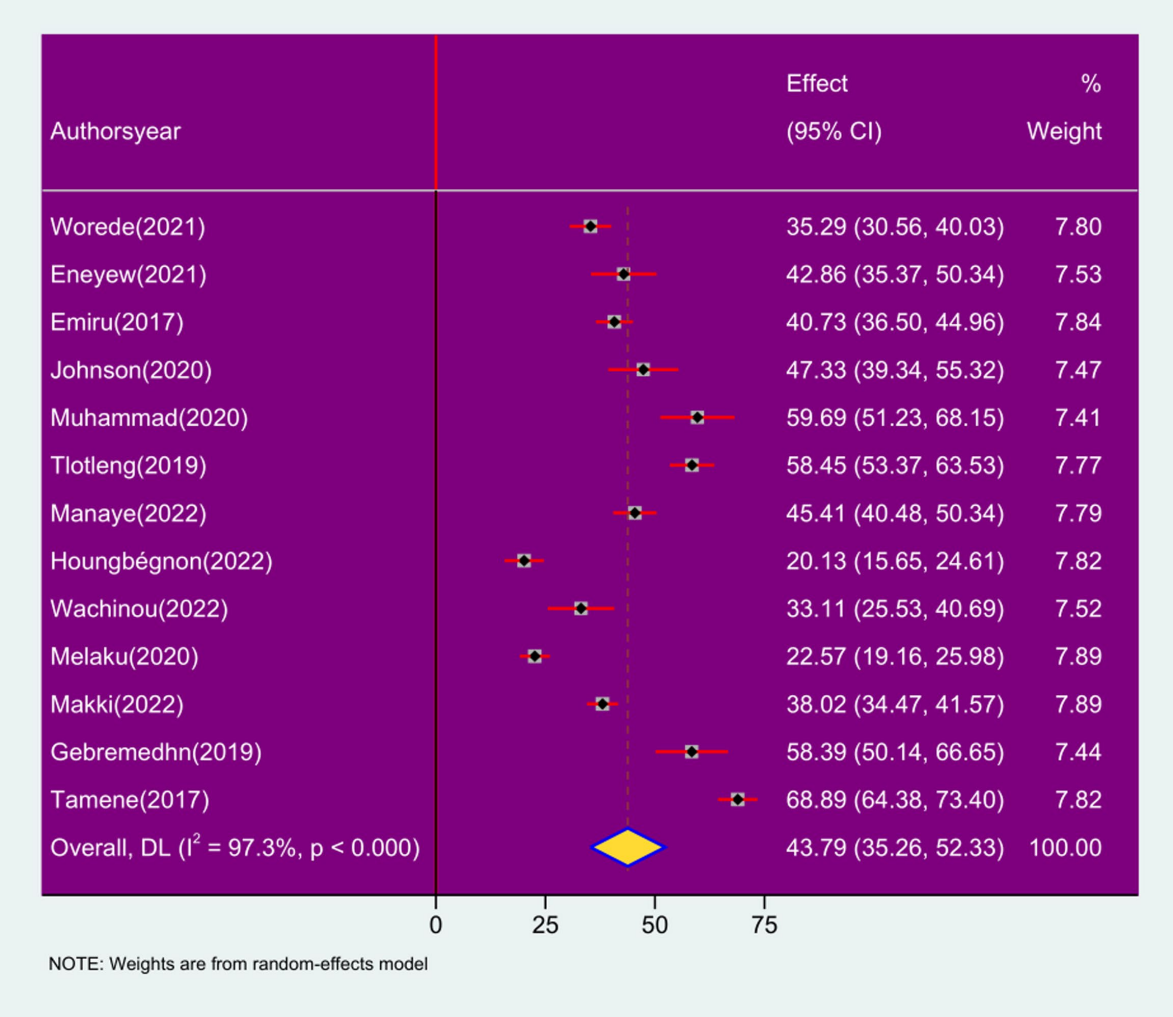
Forest plot of pooled prevalence of respiratory symptoms among sanitation workers in SSA in 2024.

The results of this study disclosed that the pooled prevalence of respiratory symptoms among sanitation workers in SSA was 43.79% (95% CI: 35.26, 52.33; *I*^2^ = 97.3%, *p* < 0.000). This figure was calculated using a random effects statistical model and is visually represented in a forest plot (Figure 2).

### Publication bias

**Figure 3:**
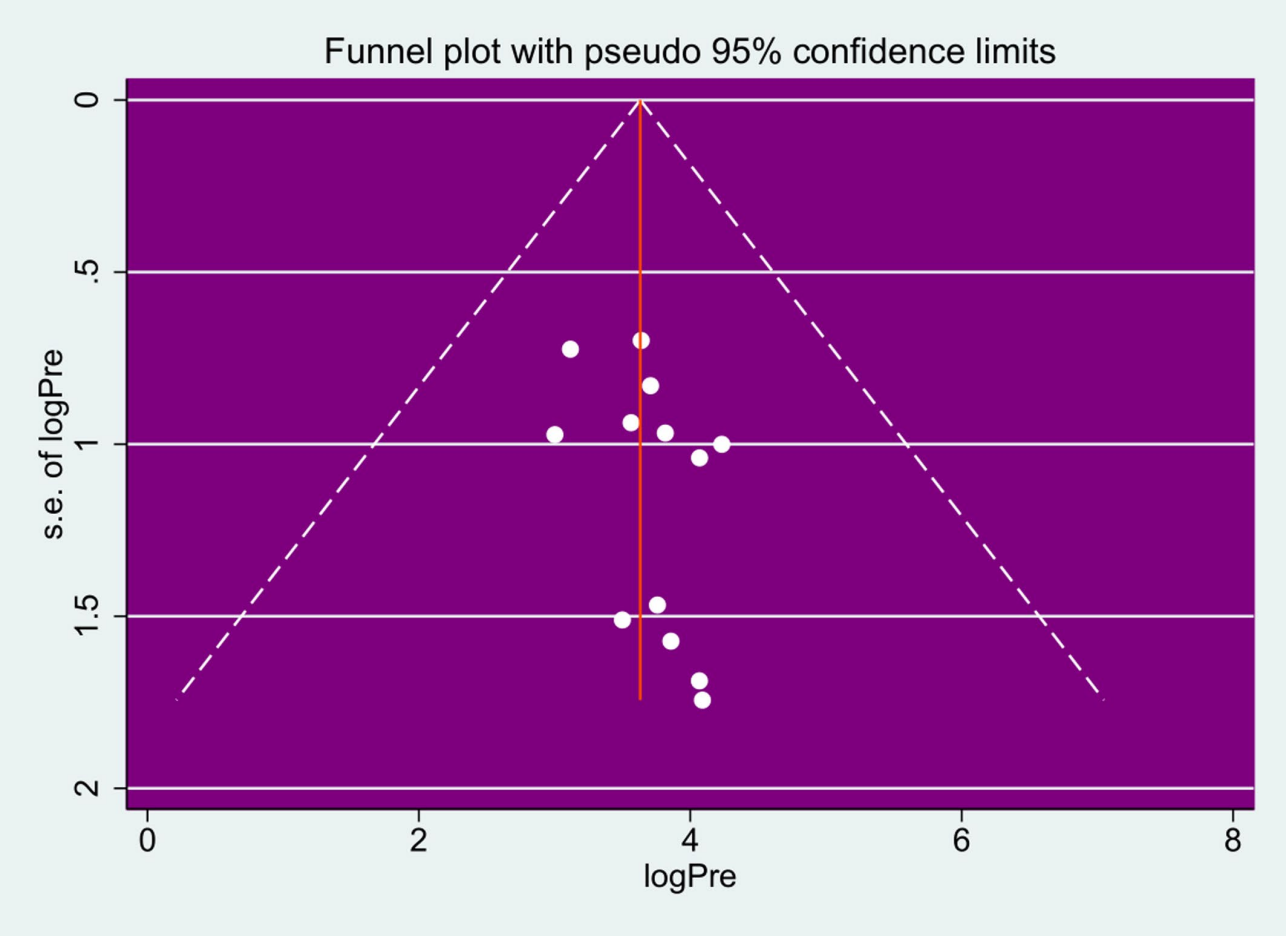
A funnel plot to test the publication bias of the meta-analysis.

The presence of heterogeneity and publication bias among the studies was assessed. The findings revealed a high level of heterogeneity among the studies included in this review (*I*^2^ = 97.3%, *p* < 0.000). To evaluate publication bias, both a funnel plot (subjective measurement) and the Egger regression test (objective measurement) were employed. The results indicated that the funnel plot was reasonably symmetrical which shows there is no publication bias among studies included in this review (Figure 3).

The finding of the funnel plot is strengthened by analyzing the Egger regression test yielding a *p* value of 0.165 which clearly indicates the absence of significant publication bias among studies included in this review (Table 2).

**Table 2:** Eggers’ regression test of studies included in the prevalence of respiratory symptoms among sanitation workers in SSA in 2024.

Std_Eff	Coef	Std.Err	*t*	*p* > *t*	[95%conf. Interval]	
Slope	3.130941	0.3509868	8.92	0.000	2.358425	3.903458
Bias	0.5136281	0.3452938	1.49	0.165	−0.2463585	1.273615

Egger’s test for small-study effects.

Regress standard normal deviate of intervention.

Effect estimate against its standard error.

Number of studies = 13 Root MSE = 0.3569.

Test of Ho: no small-study effects *p* = 0.165.

### Sensitivity analysis

**Figure 4:**
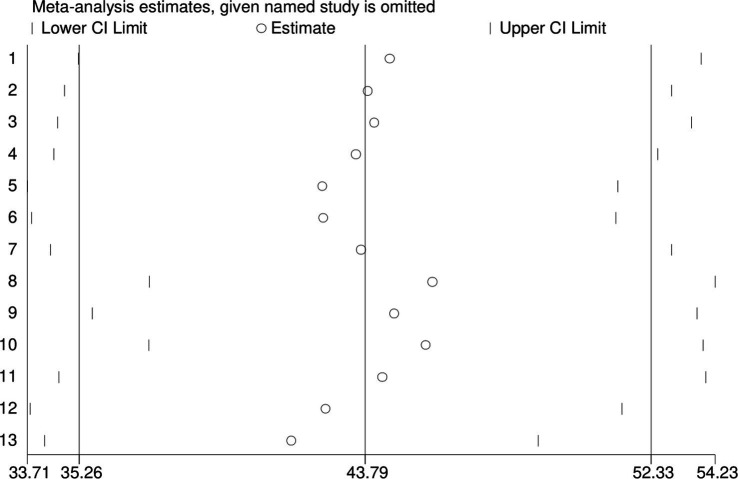
Forest plot of sensitivity analysis of the pooled prevalence of respiratory symptoms among sanitation workers in SSA.

The sensitivity analysis of the study indicated that no single study had an undue impact on the pooled prevalence of respiratory symptoms among sanitation workers in Sub-Saharan Africa. This suggests that the pooled estimate is robust and not significantly affected by any individual study included in the analysis. The fact that the pooled prevalence remained largely unchanged when any single study was removed which implies that the overall findings are stable and reliable (Figure 4).

### Sub group analysis

The subgroup analysis is important for investigating potential sources of heterogeneity among the included studies. The analysis was performed using variables study country, geographic region (East Africa, West Africa, and South Africa), sample size (below the mean and equal to or above the mean), types of sanitation tasks (municipal waste collectors vs. E-waste collectors) and publication year (prior to 2020 and in 2020 or later).

### Subgroup analysis by publication year

**Figure 5:**
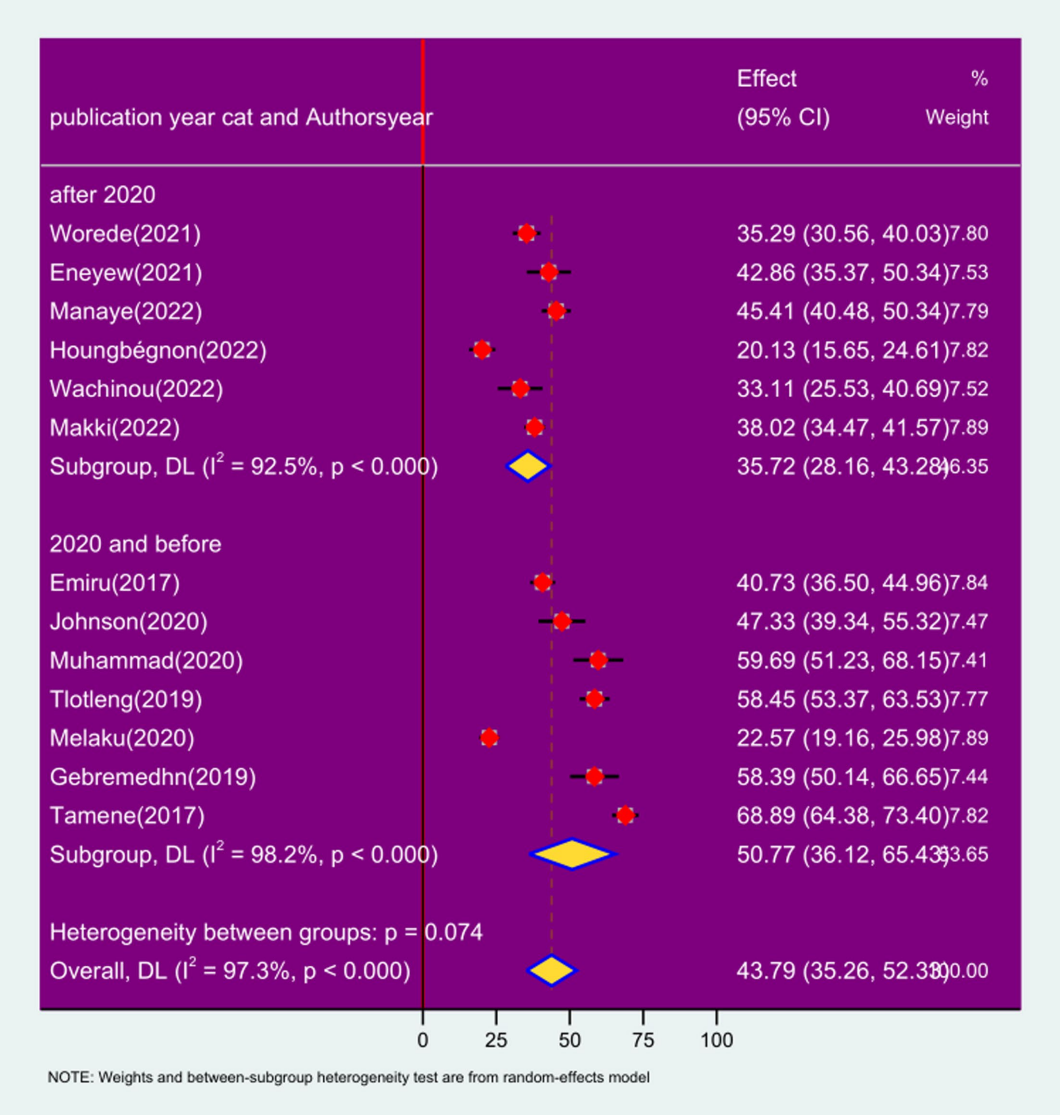
Forest plot of sub-group analysis based on year of publication on the prevalence of respiratory symptoms and associated factors among sanitation workers in SSA in 2024.

In the subgroup analysis by publication year, the prevalence of respiratory symptoms among sanitation workers in studies done after 2020 was 35.72% (95% CI: 28.16, 43.28%; *I*^2^ = 92.5%; *p* < 0.0001). On the other hand, the prevalence of respiratory symptoms among sanitation workers in studies done before 2020 was 50.77% (95% CI: 36.12, 65.43%; *I*^2^ = 98.2%; *p* < 0.0001) (Figure 5).

### Subgroup analysis by geographic region

**Figure 6:**
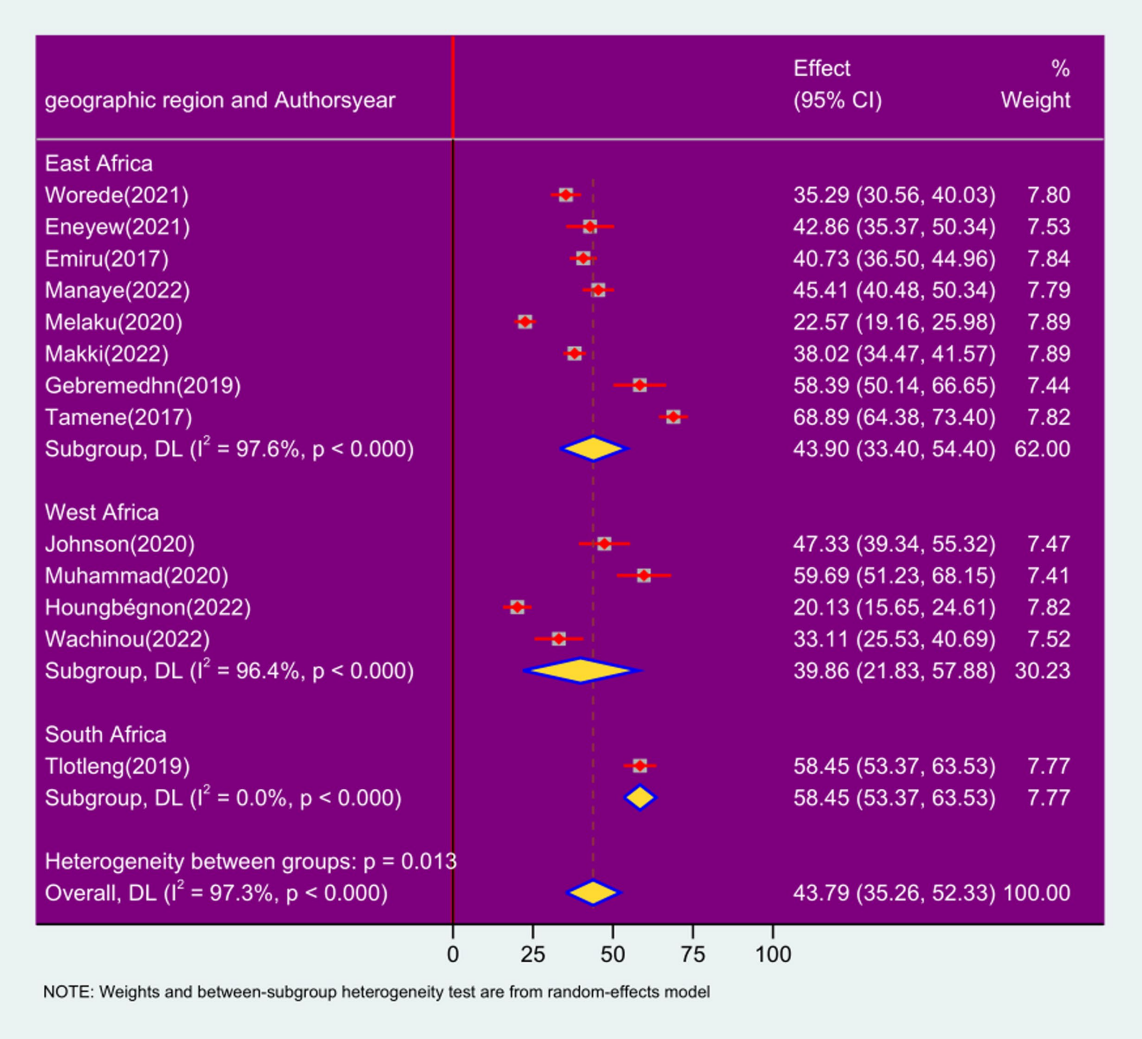
Forest plot of sub-group analysis based on geographic location on the prevalence of respiratory symptoms and associated factors among sanitation workers in SSA in 2024.

In the subgroup analysis by geographic region, the prevalence of respiratory symptoms among sanitation workers was 43.90% in East Africa (95% CI: 33.40, 54.40; *I*^2^ = 97.6%; *p* < 0.0001), West Africa 39.86% (95% CI: 21.83, 57.88); *I*^2^ = 96.4%; *p* < 0.0001, and South Africa 58.45% (95% CI: 53.37, 63.53); *I*^2^ = 0%; *p* < 0.0001 (Figure 6).

### Sub-group analysis by country

**Figure 7:**
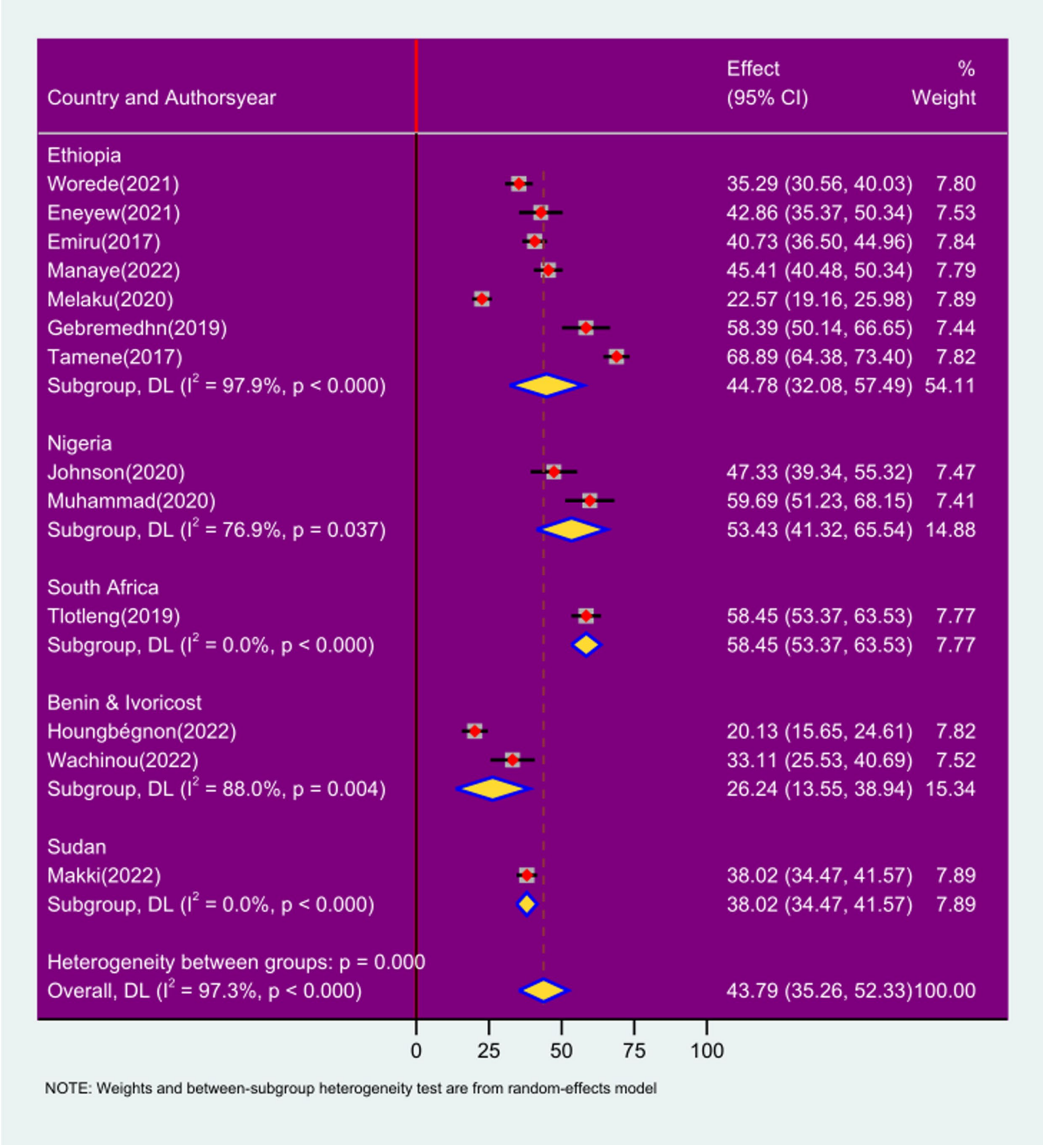
Forest plot of sub-group analysis based on country on prevalence of respiratory symptoms and associated factors among sanitation workers in SSA.

In the subgroup analysis by country, the prevalence of respiratory symptoms among sanitation workers was 38.02% in Sudan (95% CI: 34.47%, 41.57; *I*^2^ = 0.0%; *p* < 0.000), 44.78% in Ethiopia (95% CI: 32.08%, 57.49; *I*^2^ = 97.9%; *p* < 0.000), Nigeria 53.43% (95% CI: 41.32%, 65.54; *I*^2^ = 76.9%; *p* < 0.000), South Africa 58.45% (95% CI: 53.37%, 63.53; *I*^2^ = 0; *p* < 0.000), and Ivory Coast and Benin 26.24% (95% CI: 13.55, 38.94); *I*^2^ = 88.0%; *p* < 0.000 (Figure 7).

### Sub group analysis based on sample size

**Figure 8:**
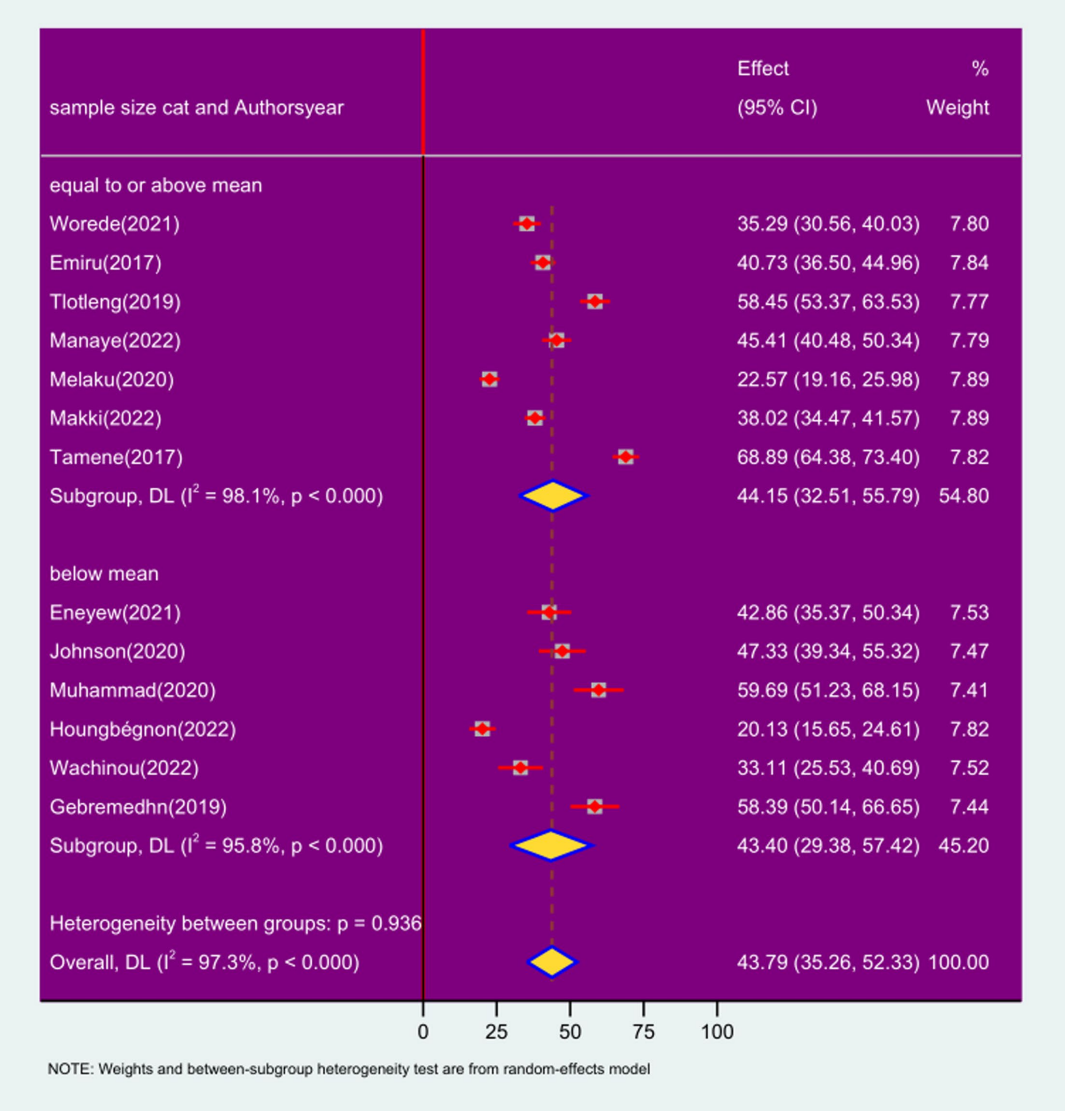
Forest plot of sub-group analysis based on sample size on the prevalence of respiratory symptoms and associated factors among sanitation workers in SSA in 2024.

The sub-group analysis based on sample size revealed that the prevalence of respiratory symptoms among sanitation workers with studies having a sample size of equal to or above the mean was 44.15% (95% CI: 32.51%, 55.79; *I*^2^ = 98.1%; *p* < 0.000), while in studies with sample size of less than the mean was 43.400% (95% CI: 29.38%, 57.42; *I*^2^ = 95.8%; *p* < 0.000) (Figure 8).

### Sub group analysis based on sanitation tasks

**Figure 9:**
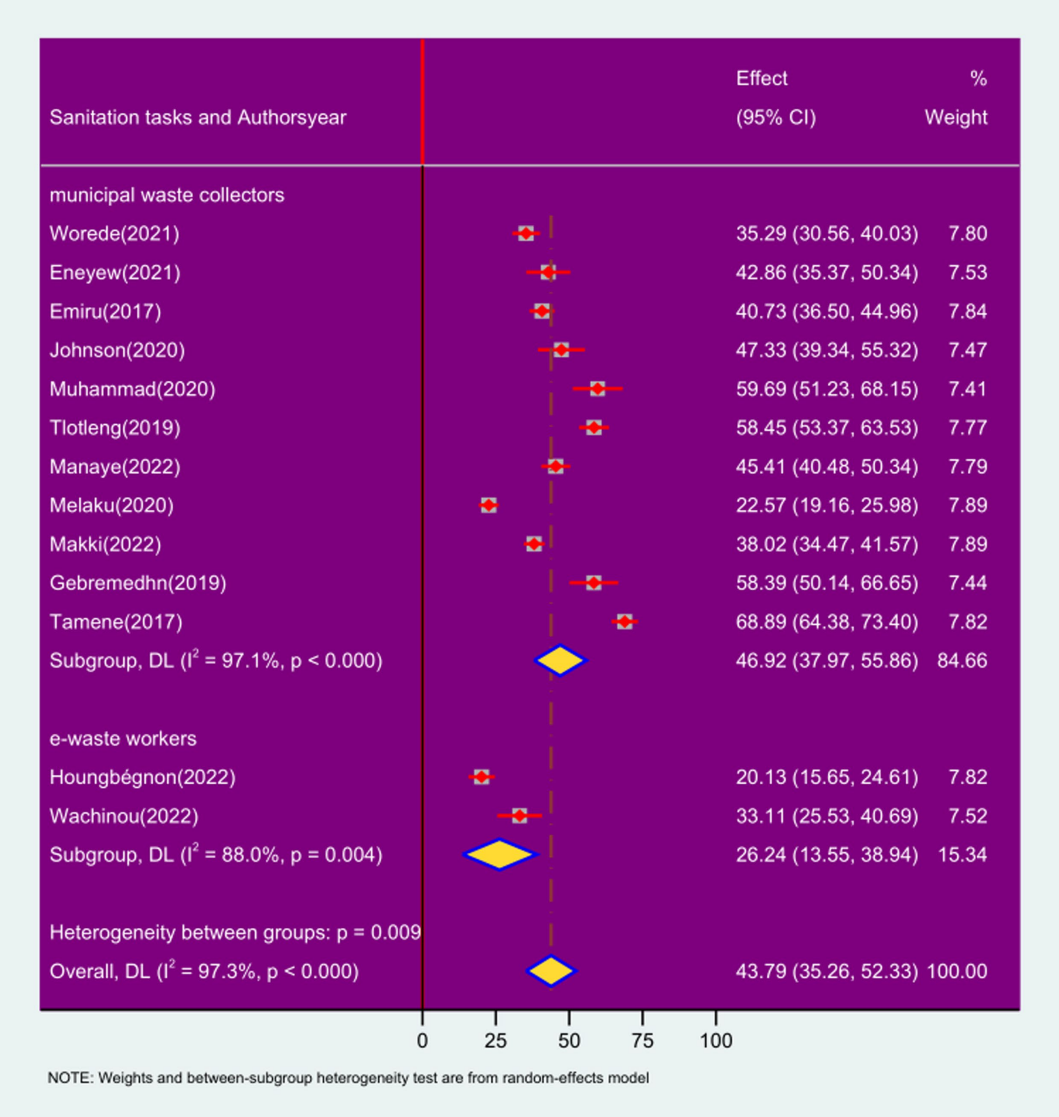
Forest plot of sub-group analysis based on types of sanitation works on the prevalence of respiratory symptoms and associated factors among sanitation workers in SSA in 2024.

The finding of this review disclosed that the pooled prevalence’s of respiratory symptoms among municipal waste collectors was 46.92% (95% CI: 37.97, 55.86; *I*^2^ = 97.1%; *p* < 0.000) whereas sanitation workers working on e = wastes have a pooled prevalence of respiratory symptom was 26.24% (95% CI: 13.55, 38.94), *I*^2^ = 88.0%; *p* < 0.000 (Figure 9).

### Meta-regression analysis

The heterogeneity assessment showed a high level of heterogeneity among studies included in this review (*I*^2^ = 97.3%, *p* < 0.001) among the included studies. Types of sanitation workers, geographic region, country category, sample size, and publication year were used to perform meta-regression analysis to find out the sources of heterogeneity. The analysis revealed that there was no statistically significant (*p* > 0.05) cause of heterogeneity in the included studies (Table 3).

**Table 3:** Univariate meta-regression analysis.

Variables	Coefficient	Std. error	95% CI	*p*-value
Types of sanitation workers	22.68449	16.37587	−9.411616, 54.7806	0.166
Publication year	−6.219072	10.61211	−27.01842, 14.58028	0.558
Geographic region	5.445116	8.506684	−11.22768, 22.11791	0.522
Sample size	−5.478972	9.203858	−23.5182, 12.56026	0552
Country	−0.3353747	4.139554	−8.448751, 7.778001	0.935

### Factors associated with respiratory symptoms among sanitation workers in SSA

**Figure 10:**
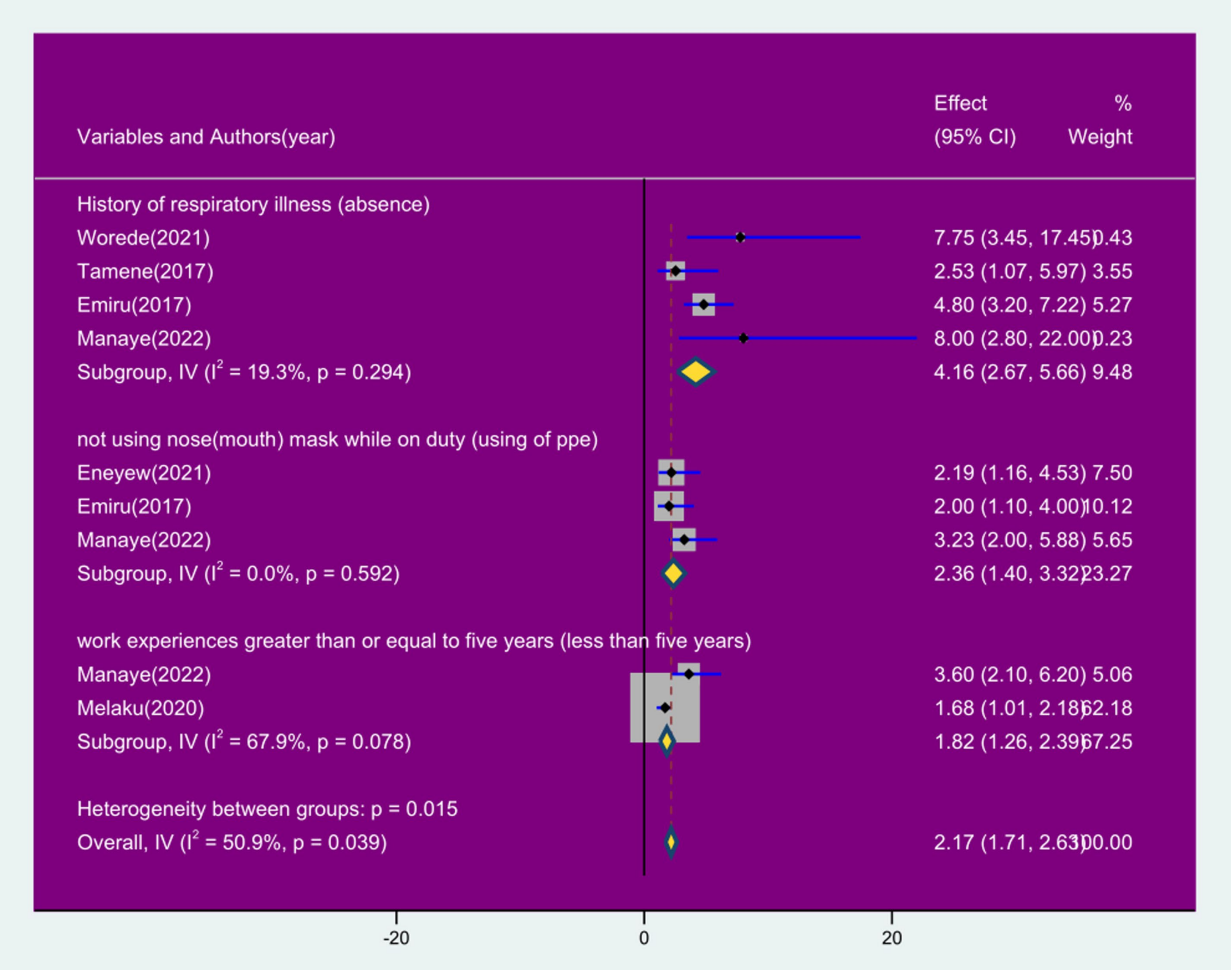
Forest plot of factors associated with prevalence of respiratory symptoms among sanitation workers in SSA in 2024.

This finding disclosed that the prevalence of respiratory symptoms was significantly associated with history of respiratory illness, lack of mask use, working experience. Six studies (7, 20, 25, 26, 30, 31) were included to assess these factors. Four studies (7, 25, 26, 30) revealed that sanitation workers with a history of respiratory illness were 4.16 times more likely to experience a respiratory symptoms compared to those without a history of respiratory illness (OR: 4.16, 95% CI: 2.67, 5.66). Additionally, three studies (20, 25, 31) also revealed that sanitation workers who did not wear masks were 2.36 times more likely to have had a respiratory symptoms compared to their counterpart (OR: 2.36, 95% CI: 1.40, 3.3.32). Furthermore, two studies (30, 31) revealed that workers with working experiences of greater than or equal to 5 years were 1.86 times more likely to experience respiratory symptoms compared to those with less than 5 years working experiences (OR: 1.81, 95% CI: 1.26, 2.39) (Figure 10).

## Discussion

Currently, the growth of the global population, increasing urbanization, rising living standards, and rapid technological advancements have all contributed to the increments of wastes both in quantity and diversity (38). Most of sanitation workers do not care for their overall health status due to low knowledge status (1). Despite the sanitation workers are increasing in dramatic way, there is an insignificant evidence or policy recommendations for their safety practices, particularly in developing countries (23). Sanitation workers face significant exposure to toxic substances and gases, making them highly vulnerable to health risks. They are also highly exposed to bioaerosols that are produced from the compost of waste may contain bacterial and fungal spores. Furthermore, they are exposed to dust and fine particles that arise from the manual handling of waste and processes such as street cleaning (4, 6, 39, 40). Occupational exposure to bioaerosols, dust, exhaust fumes and bad weather conditions play an important role in the development of respiratory problems (41). Therefore, assessing the prevalence of respiratory symptoms in these vulnerable populations lays a prominent role in designing an effective interventions.

The finding of this review revealed that the prevalence of respiratory symptoms among sanitation workers was 43.98% (95% CI: 35.53, 52.43), which was matched with studies done in Bangladesh 52.5% (42), India 45% (43), Thailand 47.4% (44). On the other hand, the finding of this review was less than studies done in Belgium 54.8% (45), Malaysia 56.8% (38), and India 61.6% (46). On the contrary, the finding of this review was higher than studies done India 11.5% (12), 32% (47), 28.6% (48), Philippines 19% (18), and Thailand 23.4% (49). The possible justification for these differences can be attributed to various factors. One key aspect is the type of waste being collected, as well as economic disparities; developed countries often possess better resources and infrastructure for waste management, which can influence the exposure levels of workers. Additionally, the length of time sanitation workers are exposed to harmful substances plays a significant role in determining health outcomes. The degree of commitment to implementing and enforcing occupational health and safety regulations, including the use of respiratory protection and personal protective equipment (PPE), can also have a substantial impact on health risks. Moreover, geographical and climatic factors can affect the frequency of respiratory symptoms. Environmental conditions in workers’ homes, such as smoking habits, the presence of pets, and the types of heating fuels utilized, may further increase the likelihood of respiratory issues among sanitation workers (42).

Personal protective equipment (PPE) is essential for preventing and managing respiratory symptoms, especially in work environments where there is a high risk of exposure to airborne pathogens and harmful materials. PPE greatly lowers the chances of inhaling these dangerous substances, making it especially vital for sanitation workers who often encounter infectious agents. However various findings disclosed that many workers do not consistently use PPE in their workplaces (16, 50), (British Journal). The finding of this review indicated that the use of face mask was one of the interventions for reducing the burden of respiratory symptoms which was supported with studies done in Gambia (8) and Bangladesh (42), and Ghana. Proper utilization of nose or mouth masks have the great potential to reduce the amount of dust inhaled, through nose and mouth masks block pathogens and dust from entering the respiratory system (16). However, several barriers to the use of PPE need to be explored, including issues related to accessibility, cost, and ease of use. Another factor contributing to the low adoption of PPE is that some workers find it uncomfortable, while others believe such equipment is unnecessary, especially in developing countries (9, 50). Furthermore, they may have low knowledge on the potential hazards in their working environment, making them more prone for injuries and other work-related illnesses (51). The effectiveness of respirators and masks depends significantly on having a proper fit. Hence, workers should be trained not only in the correct use of PPE but also in understanding the limitations of different types of equipment (52).

Sanitation workers who works for extended period time increases the risk of respiratory symptoms. A study done in China revealed that 21 potential pathogens and 15 toxic metal were detected in the aerosols wastewater (53). Various literatures reported that sanitation workers may accumulate polluted air due to an extended long period of time exposure (33). The level of dust exposure, which could also depend on years of service. Additionally, as people becomes aged, their immune system tends to weaken, making them less capable of fighting off diseases, including respiratory symptoms (49). This systematic review and meta-analysis indicated that sanitation workers who had a working experience of more than 5 years were 1.86 times more likely to experience respiratory symptoms compared to their counterparts which was supported with studies done in Egypt (54, 55), Bangladesh, Spain (56), and India (57). Particulate matter levels rise with greater intensity and duration of exposure, indicating that the impact of exposure duration diminishes over time since the last exposure and also varies with the age at which exposure first occurs (58). This may be attributed to prolonged exposure, leading to increased dust accumulation in the respiratory system.

Sanitation workers with a history of respiratory illness are more likely to experience respiratory symptoms compared to those without such a history (19, 25). Sanitation workers with a history of respiratory disorders, such as asthma or COPD, often experience chronic airway inflammation, which increases sensitivity and reactivity, making them more vulnerable to trigger respiratory impairment. History of respiratory impairment may cause to lasting lung damage, as seen in chronic bronchitis and emphysema, resulting in reduced airflow and impaired gas exchange. This decline in lung function heightens the risk of respiratory infections and exacerbates symptoms. Additionally, these individuals are more likely to develop other health issues, like cardiovascular diseases and lung cancer, complicating their respiratory health. Exposure to environmental risk factors, such as air pollution and tobacco smoke, can further affect their condition, leading to a cycle of worsening symptoms and more frequent exacerbations (22, 59, 60). The findings of this review disclosed that sanitation workers with a history of one or more respiratory illnesses were 4.16 times more likely to experience respiratory symptoms. The health consequences associated with these respiratory symptoms are significant and far-reaching. Respiratory conditions can result in diminished lung capacity, a rise in illness rates, and potentially increased mortality among sanitation workers. Examining the risk factors associated with respiratory symptoms in these marginalized populations is essential for creating effective interventions. Additionally, this research aligns with global health initiatives, including the Sustainable Development Goals (SDGs), which seek to decrease the burden of respiratory health impairments.

### Strength and limitation of the study

The strength of this review lies on its comprehensive review of existing literature, concentrating solely on studies that met the predetermined inclusion criteria to overcome selection bias. Additionally, preprints and unpublished works were excluded from the review, which may introduce methodological biases, and the results of these studies may change upon formal publication. On the other hand, this review has certain limitations. The review reliance on a limited number of databases to identify relevant literature. Additionally, this review relies on studies published in English language which may result in missing significant findings published in other languages. Another limitation of this review is the potential for recall bias in studies that depend on participants self-reporting their symptoms.

## Conclusion and recommendation

The finding of this systematic review and meta-analysis concluded that nearly half of the sanitation workers experienced respiratory symptoms. The finding of this review also stated that history of respiratory illness, lack of face mask use, and duration of working experience were factors significantly associated with the prevalence of respiratory symptoms among sanitation workers. Subgroup analysis was conducted based on publication year, geographic region, country, types of sanitation workers, and sample size and no sources of heterogeneity was detected. The prevalence of respiratory symptoms in studies done after 2020 was reduced to one-third. Variations in prevalence of respiratory symptoms were also noted by country, with South Africa recorded the highest rates whereas the lowest rate was recorded in Ivory Coast with only one-fifth of workers exhibited the problem. Hence, awareness creation should be done on the use of personal protective equipment (PPE) and awareness of occupational hazards to improve health literacy and empower workers. Promotion of improved working environments, such as adequate ventilation, to minimize exposure to harmful substances should be done. Additionally, establishment of regular health screenings to track respiratory health and enable them to take early intervention. Furthermore, ensuring all sanitation workers have access to effective PPE, with consistent evaluations to ensure its effectiveness. Finally, concerned governmental and non-governmental organizations should formulate policies that appreciate the role of sanitation workers and provide essential funding to enhance their health and safety.

## Data Availability

The original contributions presented in the study are included in the article/supplementary material, further inquiries can be directed to the corresponding author.
